# Knowledge, attitude, practices, and perceived barriers to using point-of-care ultrasound by Asian primary care physicians – a mixed method study

**DOI:** 10.1186/s12913-024-11865-5

**Published:** 2024-11-05

**Authors:** Amy Pui Pui Ng, Kiki Sze Nga Liu, Zoey Cho Ting Wong, Zoe Ho Wai Tang, Eric Yuk Fai Wan, Esther Yee Tak Yu, Man Chi Dao, Chun Yu Wu, Tai Pong Lam

**Affiliations:** 1 Department of Family Medicine and Primary Care, The University of Hong Kong-Shenzhen Hospital, 1 Haiyuan 1st Rd, Futian District, Shenzhen, Guangdong Province 518009 China; 2 Department of Family Medicine and Primary Care, School of Clinical Medicine, Li Ka Shing Faculty of Medicine, The University of Hong Kong, Hong Kong SAR, China; 3 Department of Pharmacology and Pharmacy, LKS Faculty of Medicine, 2/F, Laboratory Block, HKU, 21 Sassoon Road, Pokfulam, Hong Kong SAR China; 4 Department of Family Medicine and Primary Health Care, Kowloon West Cluster, Hospital Authority, Hong Kong SAR, China

**Keywords:** Ultrasound, Diagnostic imaging, Primary health care, Education

## Abstract

**Background:**

Although research shows that point-of-care ultrasound is helpful in primary care, there is little research on point-of-care ultrasound use and the barriers to its use in Asia. This study estimated the prevalence of primary care physicians using point-of-care ultrasound in Hong Kong and assessed their perceived knowledge, attitude, practices, and barriers to using point-of-care ultrasound.

**Study design:**

This was a mixed-method study: cross-sectional survey, followed by semi-structured interviews. Primary care physicians who were members of the Hong Kong College of Family Physicians and/or were clinical teachers affiliated with the Department of Family Medicine and Primary Care at the University of Hong Kong were invited to participate.

**Results:**

A total of 330 and 14 completed the survey and interviews, respectively. The prevalence of respondents using point-of-care ultrasound was 22.5%. Perceived knowledge was fair (mean score: 1.9 out of 4; SD: 0.6). The attitudes were mostly positive (mean score: 3.0 out of 4; SD: 0.5). Majority stated that barriers to using point-of-care ultrasound were related to training (90.9%), the competence of point-of-care ultrasound skills (90.2%), and clinical support (89.5%). Qualitative data identified that most participants found point-of-care ultrasound useful; however, participants felt that its use was limited by their competence of point-of-care ultrasound and by factors related to their clinical practice.

**Conclusions:**

Almost a quarter of respondents are using point-of-care ultrasound with a majority having positive attitudes. They lack confidence in their skills as knowledge is poor but simultaneously find training and clinic support limited.

**Supplementary Information:**

The online version contains supplementary material available at 10.1186/s12913-024-11865-5.

## Background

Point-of-care ultrasound (POCUS) refers to ultrasound done at the bedside [1]. Globally, POCUS used by primary care physicians (PCP), including family physicians and general practitioners, is becoming more popular [2] with the prevalence of PCPs using POCUS varying from 1% in Europe [3] to 76% in rural Canada [4]. There are many potential benefits for POCUS use in primary care. Studies show that POCUS in primary care can supplement physical examinations [5] in under 10 min [6, 7]. A study in Denmark found that POCUS used by PCPs entailed a change in diagnoses in 49.4% of patients, helped to increase confidence in a diagnosis in 89.2% of patients, and resulted in a change in the management plan for 50.9% of patients, including a reduction in intended referrals to secondary care by half [6]. POCUS, in this study, altered the GPs’ clinical decision-making in nearly three out of four consultations. POCUS use also has the potential to reduce overall health care costs [8] by reducing referrals to secondary care and reducing the need for formal imaging which would require technicians to scan and radiologists to interpret and report. Lastly, it helps with timely diagnosis which can improve patient outcomes and lessen costs by using POCUS as a triage tool [9]. 

In addition, PCPs’ attitudes towards POCUS can affect its use in practice. Research shows that PCPs’ and patients’ perceptions of POCUS are positive which is encouraging for its widespread adoption [10, 11]. Despite the benefits, barriers to using POCUS in primary care include a lack of training, time to perform, confidence in interpreting images, financial support, and ultrasound equipment [12–15]. Although misdiagnoses are not uncommon [10], evidence supports that POCUS is safe [16] and has limited implications on malpractice lawsuits [17]. A systematic review of 51 articles showed that PCPs use POCUS for a variety of body systems including the cardiovascular system, abdominal, gynecological and obstetrical areas, and musculoskeletal system for both explorative and focused scans [8]. However, it is clear that focused examinations that are used to answer a specific clinical question such as “If a 40-year-old female with lower abdominal pain has an ectopic pregnancy?” were more accurate than explorative examinations such as “What are the potential causes of abdominal pain in a 40-year-old female?” [8] It is unclear whether PCPs are aware that the former (i.e., performing focused examinations to answer specific clinical questions) is often considered the goal when using POCUS in clinical practice [18]. 

Studies support that PCPs can be trained to use ultrasound [10]. In North America and Europe, POCUS training in Family Medicine (FM) residency has been rapidly expanding but incorporating POCUS into the medical curriculum in Asia has just begun [1, 3, 12, 14, 18]. In 2021, The University of Hong Kong began ultrasonography training in the undergraduate medical curriculum after a successful pilot study [19]. Although trainees in other post-graduate specialties have ultrasound training in Hong Kong, the topic is absent in the post-graduate training programs in FM with few trainees being exposed to POCUS in hospital-based rotations and primary care clinics. Also, practicing PCPs have limited choices in training programs. The Hong Kong College of Family Physicians (HKCFP) offers introductory POCUS courses; but, these are sporadic. Local options for comprehensive courses include a two-year part-time Master’s program by The Chinese University of Hong Kong, a program by the Hong Kong Institute of Musculoskeletal Medicine, or self-hiring sonographers and physicians to learn from.

Moreover, although PCPs are performing POCUS in Hong Kong, studies on the prevalence, knowledge, attitude, practices, and barriers of PCPs using POCUS in Hong Kong and Asia are lacking. Like other Asian countries, Hong Kong has an active private sector, especially for primary care, that runs parallel with the public sector and previous research supports that private Hong Kong doctors are more likely to undertake postgraduate training for different reasons than public doctors [20]. Also, most POCUS-related studies in primary care utilized surveys only, and prior research suggested it was difficult to assess a complete catalog of barriers by analyzing surveys only [21]. Therefore, a mixed-method study combining qualitative interviews and surveys would help broaden the understanding of barriers, while sampling both private and public sectors. The findings will help to guide the profession and healthcare institutions to overcome the barriers to using POCUS such as determining how to best incorporate POCUS training and clinical support. The overall implication will be to strengthen PCPs’ ability to accurately and efficiently diagnose health problems by enabling more POCUS which will be a cost-effective method to improve patient-care.

Thus, this study aims to understand Hong Kong PCPs’ current practices in using POCUS; to investigate their knowledge of POCUS; to understand their attitude towards using POCUS; and to understand their perceived barriers to learning and using POCUS.

## Methods

### Study design and participants

This was a mixed-methods study: a cross-sectional survey, followed by semi-structured interviews.

#### Cross-sectional survey

Based on an extensive literature search [10, 12, 21–23] on similar surveys, questions related to the research aims were modified and adapted to the Hong Kong primary care system (Additional file 1). Content validity of the questionnaire was done first involving six experts and a second evaluation by seven PCPs. The Average Scale Content Validation Index for relevance was 0.98 indicating a content-valid scale [24]. Cronbach’s Alpha for the “attitude”, “knowledge” and “barrier” domains were 0.889, 0.922, and 0.797 respectively, which exceeds the cutoff for adequate internal reliability (≥ 0.7).

The survey was divided into five sections: (1) Demographic data was obtained about the PCPs’ training experience in POCUS, gender, age, type of practice, and postgraduate qualifications; (2) Information about current practices was asked, including an assessment on POCUS device availability and whether PCPs had used POCUS in primary care and their practice; (3) Perceived knowledge of POCUS was ascertained by eight knowledge items that were rated on a 4-point Likert Scale (“inadequate” to “excellent”); (4) Attitude of POCUS were assessed from 11 attitude items that were rated on a 4-point Likert Scale (“strongly disagree” to “strongly agree”). There was one question on interest to POCUS; (5) Barriers to POCUS were assessed with thirteen statements that were assessed on a 4-point Likert Scale (“not an important barrier at all” to “very important barrier”).

Members of the HKCFP and/or clinical teachers affiliated with the Department of Family Medicine and Primary Care from The University of Hong Kong with valid email addresses were invited twice via email between January – February 2022. In calculating the available population, we excluded HKCFP members who lived outside of Hong Kong, retired, allied health professionals and medical students, and clinical teachers who were already members of the HKCFP (to prevent duplicate counting). The online questionnaire was voluntary and self-administered via Qualtrics if the doctors met the inclusion criteria such that they were currently working in primary care in Hong Kong or were enrolled as FM trainees. Participants agreed to an online consent form and were offered a HK$50 (US$6.5) coffee voucher.

For the sample size calculation, as the prevalence in Hong Kong is unknown, we assumed the proportion of PCPs using POCUS is 30% based on the prevalence of PCPs using POCUS found in more population-dense areas of Europe ranging from 1–47% [3]. The required sample size for a population of around 1584 PCPs in Hong Kong is 269 with a margin of error being 5% and a confidence level of 95%.

#### Semi-structured interviews

The topic guide was developed for the semi-structured interviews based on the analysis of the survey data and included questions about the PCPs’ practices, how they order an urgent ultrasound, what they know about POCUS in FM, what benefits, problems, and barriers they have in using POCUS and suggestions on how to overcome the barriers (Additional file 2). Purposeful samplings of PCPs ensured that PCPs came from different service institutions and had different experience levels and interests to POCUS. Invitation letters were sent by email to specific clinical teachers of the Department and HKCFP members who were eligible for the survey, which included representatives from the HKCFP POCUS interest group, FM vocational trainees, and members of the Medical Licentiate Society of Hong Kong, a group of foreign-trained doctors with medical license to work in Hong Kong, from public and private settings. Interviews were conducted until data saturation was achieved. Fourteen PCPs were interviewed between May and July 2022 without repeat interviews. There were no refusals among all participants. Five participants were acquainted with the interviewer as a fellow vocational trainee or teacher in the Department but not at a personal level; whereas the others were never acquainted with the interviewer. Participants knew where the researcher worked and the purpose of the research. No specific characteristics were reported about the interviewer to the participants. Participants signed a consent form and received a HK$200 (US$25) coffee voucher. They were audio recorded over Zoom platform. The interviews lasted 30 min to one hour and were carried out in English and/or Cantonese and no field notes were taken. No non-participants were present during the interviews. The interviews were conducted by a female family physician (AN), a Clinical Assistant Professor with formal training in qualitative research and prior experience in conducting qualitative interviews and analysis.

### Analysis

#### Quantitative analysis

Survey results were extracted from Qualtrics. Missing data was excluded from analysis. Valid data was coded and imported into SPSS version 26.0 for analysis.

Descriptive analysis was performed for socio-demographic characteristics, POCUS usage, attitudes toward POCUS, perceived knowledge of POCUS, and barriers to POCUS. Two questions were reverse-coded under “attitude” before analysis, with higher scores indicating more positive attitudes.

For attitude and barrier domains, survey items were grouped under subgroups for focused interpretation. The survey items included in each subgroup are displayed in Additional file 3. Mean of scores for each subgroup were calculated. For “attitude” subgroups, responses were categorized as “negative” (mean score ≤ 2.5) or “positive” attitudes (mean score > 2.5 (out of 4)), in which 2.5 refers to the median of the 4-point scale. Similarly, each subgroup of “barriers” was categorized as “disagree” (mean score ≤ 2.5) and “agree as barriers” (mean score > 2.5 (out of 4)). Frequencies and proportions for each group were calculated. “Knowledge” was presented by mean and standard deviation (SD). Overall mean scores were presented for the three domains. The association of attitude subgroups with POCUS usage in the past 12 months and interest in POCUS were assessed by Chi-square tests. The mean of knowledge scores for each category of POCUS usage in the past 12 months and interest in POCUS were also compared by t-test. The relationship between “knowledge”, “attitude”, “interest” and “usage” with “interest” and “usage” was evaluated by logistic regression, which was adjusted for gender, years after graduation from medical school, country of graduation, and type of service institutions to reduce potential selection bias. *P* < 0.05 was defined as statistically significant for all statistical tests.

#### Qualitative analysis

Transcripts were transcribed verbatim and translated to English (if applicable) by two researchers (KC, ZT) and the accuracy was checked by a third researcher (AN). The transcripts were not returned to the participants. A thematic approach was used to develop codes from the data (e.g. help to make an earlier diagnosis), and group them into subthemes (e.g. Improving diagnosis and management) and themes (e.g. Usefulness of POCUS). AN and ZT coded the transcripts using NVivo software. The reliability and validity of the analysis and interpretation were assessed by checking the coding consistency between the two sets. Discrepancies were settled by discussion with the two coders and a third researcher (KL) to reach an agreement on a common theme. Participants did not provide feedback on the analysis. KC, a female teaching assistant with a bachelor’s degree, has experience in transcription and translation. ZT, a female research assistant with a bachelor’s degree, has experience in transcription, translation, and qualitative analysis. KL, a female PhD candidate, with extensive experience in qualitative analysis, completed her PhD one year later.

## Results

### Quantitative survey

**Fig. 1:**
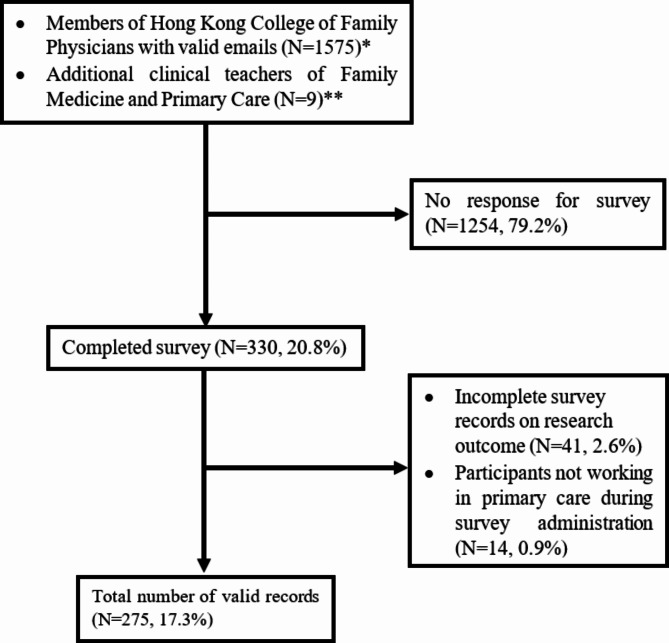
Flow chart of subject recruitment. *Members of the Hong Kong College of Family Physicians with valid email address excluding those living out of Hong Kong (63), retired (29), allied health professionals (17) and medical students (64). ** Additional clinical teachers of Family Medicine and Primary Care from The University of Hong Kong (included only the non-family medicine doctors as those with family medicine training be already included as a members of the Hong Kong College of Family Physicians)

The response rate was 20.8%. After removing incomplete, ineligible, and duplicate records, 275 (17.3%) surveys were included for analysis as shown in Fig. 1. Baseline characteristics of the patients are shown in Table 1. The majority of respondents were Hong Kong graduates (83.3%), and the mean (SD) years after graduation was 21.3 (11.3). Table 2 shows the descriptive results for POCUS knowledge, attitude, interest, practices, and barriers. Participants have completed all items except for interest (*n* = 250, 90.9%).

**Table 1:** Socio-demographic characteristics of participants

Factors (count, %, *N* = 275)		
**Age** (mean (SD)), year (*n* = 235)^1^	44.8 (11.0)	
**Gender**		
Male	156	56.7%
Female	108	39.3%
Prefer not to say	11	4.0%
**Years after graduation from medical school** (mean (SD)), year (*n* = 274)^1^	21.3 (11.3)	
**Country of graduation**		
Hong Kong	229	83.3%
Australia, Canada, The UK, The USA	28	10.2%
Mainland China	9	3.3%
Others^2^	9	3.3%
**Postgraduate qualification** ^3^		
FHKAM (FM)	129	46.9%
FRACGP	132	48.0%
FHKCFP	131	47.6%
Diploma in Family Medicine	88	32.0%
Currently undergoing higher training in Family Medicine	7	2.5%
Currently undergoing basic training in Family Medicine	23	8.4%
No postgraduate qualification in Family Medicine	7	2.5%
Others^4^	31	11.3%
**Service institution** (*n* = 269)^1,5^		
Public institutions	151	56.1%
Private institutions	112	41.6%
Both public and private institutions	6	2.2%

*FRACGP *Fellowship of the Royal Australian College of General Practitioners, *FHKCFP *Fellowship of Hong Kong College of Family Physicians, *FHKAM (FM) *Fellow of the Hong Kong Academy of Medicine (Family Medicine), *SD *Standard deviation. The results were presented as mean (SD) or count and %, as appropriate

^1^Missing data and non-specific answers were excluded from calculation

^2^Others: Taiwan, Myanmar

^3^Multiple selection of options is allowed

^4^Others: Non-Family Medicine post-graduate qualifications (e.g. Surgery, Medicine, Psychiatry, Emergency Medicine), Family Medicine-related post-graduate qualifications from overseas (e.g. MRCGP) and other diplomas (e.g. Child health, dermatology)

^5^Public institutions: Department of Health, Hospital Authority and Others (including non-governmental organization and university); Private institution: Solo private clinic, Group private practice and Private hospital; Both public and private institutions: participants provide multiple answers, with both public and private institutions included

**Table 2:** Descriptive results of POCUS knowledge, attitude, interest, practices and barriers

**Knowledge** (Maximum score = 4)^3^ (*N* = 275)					Mean (SD)
Indications for POCUS					2.2 (0.9)
Anatomy and Pathology					2 (0.7)
Physics of ultrasound, using the machine, and choosing different modes of imaging					1.8 (0.8)
Interpretation of ultrasound images and documentation of POCUS image and report					1.6 (0.7)
Overall mean score^2^					1.9 (0.6)
**Attitude** (Maximum score = 4) (count, %, n, *N* = 275)	Negative attitude^1^		Positive attitude^1^		Mean (SD)
On POCUS training	56	20.4%	219	79.6%	3.1 (0.6)
On clinical usefulness of POCUS	21	7.6%	254	92.4%	3.1 (0.5)
On harmfulness of POCUS	58	21.1%	217	78.9%	2.9 (0.6)
On cost-effectiveness of POCUS	58	21.1%	217	78.9%	2.9 (0.7)
On patient preference on provider	133	48.4%	142	51.6%	2.5 (0.7)
Overall mean score^2^					3.0 (0.5)
**Interest** in using POCUS in current practice (count, %, n, *N* = 250)					
Yes	179	71.6%			
No	25	10.0%			
Not sure	46	18.4%			
**Practices** (count, %, n, *N* = 275)					
Has used POCUS in the last 12 months in their clinical practice	62	22.5%			
Current practice location has a POCUS device on-site					
Yes	102	37.1%			
No	166	60.4%			
Not sure	7	2.5%			
Has received POCUS training in the past	111	40.4%			
**Barriers** (Maximum score = 4) (count, %, n, *N* = 275)	Disagree as barrier^1^		Agree as barrier^1^		Mean (SD)
Competence of POCUS skills	27	9.8%	248	90.2%	3.2 (0.6)
Training support	25	9.1%	250	90.9%	3.2 (0.5)
Clinical support	29	10.5%	246	89.5%	3.2 (0.6)
Financial concerns	81	29.5%	194	70.5%	2.9 (0.7)
Clinical usefulness	164	59.6%	111	40.4%	2.3 (0.8)
Overall mean score^2^					3.1 (0.4)

*POCUS *Point-of-care ultrasound, *SD *Standard deviation

^1^‘Negative attitude’ and ‘Disagree as barrier’ refer to mean score ≤ 2.5; ‘Positive attitude’ and ‘Agree as barrier’ refer to mean score > 2.5

^2^‘Overall mean score’ refers to the mean of average scores over all the survey questions under particular category (Attitude,knowledge or barrier),which ranged from 1 to 4

^3^Knowledge is defined as continuous variable. Only mean and SD are displayed

The overall mean (SD) score of “knowledge” domain was 1.9 (0.6) of 4 and interpretation of ultrasound images and documentation of POCUS images and reporting were the most deficient areas (mean score (SD) 1.6 (0.7) of 4). Majority of the participants (> 78.9%) had positive attitudes in all the subgroups except for patient preference on the provider of ultrasound where 48.4% of respondents thought radiologists would be preferred over PCPs. Over two-thirds (*n* = 179, 71.6%) of respondents were interested in using POCUS in their practice. The prevalence of POCUS usage in the past 12 months was 22.5% (*n* = 62). Over one-third (*n* = 102, 37.1%) of participants had access to POCUS devices in their current practice locations, and 40.4% (*n* = 111) previously received POCUS training. A majority considered the training support of POCUS as the highest rated barrier (*n* = 250, 90.9%) followed by barriers for competence of POCUS skills (*n* = 248, 90.2%) and clinical support of POCUS (*n* = 246, 89.5%). The frequency and proportion of respondents for each survey items under domain “knowledge”, “attitude” and “barrier” are shown in Additional file 4.

#### Association of “knowledge” and “attitude” with “interest” and “usage”

As shown in Table 3, knowledge was correlated with both interest in POCUS and POCUS usage in the past 12 months with significance (*p* < 0.001). All subgroups of attitude were associated significantly with interest in POCUS (*p* < 0.001) whereas only the attitude of patient preference on provider had a statistically significant relationship with POCUS usage in the past 12 months (*p* = 0.044).

The adjusted odds ratio (OR) estimates can be found in Additional file 5. Respondents who had a positive attitude towards POCUS training and a higher knowledge score were more likely to express interest in POCUS (OR: 4.086 [95% CI 1.897, 8.8]) and report using POCUS within the past 12 months (OR: 6.511 [95% CI 3.517, 12.053]), respectively.

**Table 3:** Correlation of attitude and knowledge with POCUS usage and interest in POCUS

	Interest in POCUS (*N* = 275)			POCUS usage within past 12 months (*N* = 275)		
	No	Yes	*p*-value	No	Yes	*p*-value
**Knowledge**	1.6 ± 0.5	1.9 ± 0.6	< 0.001*	1.7 ± 0.5	2.4 ± 0.7	< 0.001*
**Attitude**						
On POCUS Training			< 0.001*			0.893
Negative	33 (12.0%)	23 (8.4%)		43 (15.6%)	13 (4.7%)	
Positive	38 (13.8%)	181 (65.8%)		170 (61.8%)	49 (17.8%)	
On clinical usefulness of POCUS			< 0.001*			0.885
Negative	14 (5.1%)	7 (2.5%)		16 (5.8%)	5 (1.8%)	
Positive	57 (20.7%)	197 (71.6%)		197 (71.6%)	57 (20.7%)	
On harmfulness of POCUS			< 0.001*			0.744
Negative	25 (9.1%)	33 (12.0%)		44 (16.0%)	14 (5.1%)	
Positive	46 (16.7%)	171 (62.2%)		169 (61.5%)	48 (17.5%)	
On cost-effectiveness of POCUS			< 0.001*			0.165
Negative	29 (10.5%)	29 (10.5%)		41 (14.9%)	17 (6.2%)	
Positive	42 (15.3%)	175 (63.6%)		172 (62.5%)	45 (16.4%)	
On patient preference on provider			< 0.001*			0.044*
Negative	48 (17.5%)	85 (30.9%)		110 (40.0%)	23 (8.4%)	
Positive	23 (8.4%)	119 (43.3%)		103 (37.5%)	39 (14.2%)	

*POCUS *Point-of-care ultrasound, *SD *Standard deviation

*Significance at *p*-value < 0.05

### Qualitative semi-structured interviews

Of the 14 interviewees, 4 used POCUS in their practice, 9 worked in the public setting, and 10 graduated from Hong Kong medical schools. Three major themes were discovered: Usefulness of POCUS, competence of POCUS skills, and limitations in using POCUS in clinical practice. Details are elaborated with illustrative quotes under each subtheme and additional quotes are listed in Additional file 6.

#### Theme 1: usefulness of POCUS

Participants agreed that POCUS was useful to improve diagnosis and management by helping to make an earlier and more accurate diagnosis, determine the next steps of care, perform procedures, and improve patient communication.

“I regard POCUS as an extra pair of hands, or extra pair of eyes, with x-ray vision, or more sensitive hands, to help the patient in more depth through the physical exam.” *(Participant 10 – Private solo*,* POCUS user*,* 26 years post HK grad)*.

Participants commented that it helped to reduce burdens on patients by saving patients’ time and money and protecting them from harmful investigations.

“[POCUS] makes the whole patient experience smoother, avoids an unnecessary trip to the Imaging Center and … reduces the cost for the patient as well.” *(Participant 3 – Private group practice*,* non-POCUS user*,* interested in POCUS use*,* 13 years post UK grad)*.

POCUS was also useful in reducing burdens on the healthcare system by reducing the need for other imaging and referrals to secondary care.

“[POCUS] will lessen the burden of referral to emergency departments, if some emergency condition can be ruled out by point-of-care ultrasound.” *(Participant 8 – Public group*,* POCUS user*,* > 10 years post HK grad)*.

#### Theme 2: competence of POCUS skills

Participants reported difficulties in improving their POCUS skills, which were related to the barriers to training such as a lack of time for POCUS training, official accreditation in POCUS, training courses, and clinical support (such as financial support and supervision).

“The problem of supervision. Because you know, training… you need someone to supervise the trainees to do [POCUS]. I perceived some barriers and maybe some obstacles because not many senior family physicians have acquired this skill yet.” *(Participant 6 – Public group*,* POCUS user*,* 19 years post HK grad)*.

Some participants lacked the confidence to obtain competent POCUS skills fearing that it could lead to harm for both patients and doctors including medico-legal problems.

“Some kind of like, liver adenoma sort of things, those things are benign… but medicolegally if you miss it, you still have some problem. Because if you just say, “Okay you’re fine, you don’t need that follow-up”, but probably a half year later, or one year later, the patient has a formal ultrasound, and it would be found to have a lot of these lesions, [then] you still can be in trouble.” *(Participant 9 – Public group*,* POCUS user*,* 20 years post HK grad)*.

Some perceived that radiologists are better skilled at ultrasound and able to issue formal ultrasound reports thus ultrasound done by PCP would be inferior. In addition, participants felt patients had the same perceptions.

“Nowadays patients are more knowledgeable, they know the difference between a family physician and also radiologist. They know that family physician…may not be expert of ultrasound findings, so they will feel more confident or more reassuring, to go for a proper ultrasound done by the radiologist.” *(Participant 8 – Public group*,* POCUS user*,* > 10 years post HK grad)*.

#### Theme 3: limitations in using POCUS in clinical practice

The majority of the participants stated that the practical limitation of insufficient time to use POCUS in their consultations is a concern, and if they were to use POCUS, there would be an opportunity cost.

“I don’t think we have enough time to do the [POCUS] in this packed clinical schedule. So… I think if we really use this ultrasound, they need to have a special session arranged, so we see fewer patients and more time to… do the ultrasound and explain the report to the patient.” *(Participant 1 – Public group*,* non-POCUS user*,* interested in POCUS use*,* 5 years post HK grad)*.

Few had concerns about the high cost of the POCUS machine, the large space it may take up, and issues regarding maintenance of the machine.

“The clinic space in Hong Kong is very limited. And even within the consultation room or in the treatment room, finding a space to house this machine that might not be very frequently used, sometimes could be difficult.” *(Participant 3 – Private group*,* non-POCUS user*,* interested in POCUS use*,* 13 years post UK grad)*.

Some also believed there is a lack of compelling need for POCUS in their current practice as most cases encountered by the PCPs did not need POCUS and when needed, private ultrasound could be completed promptly such that POCUS contributed little to the management.

“But for patients who are not so urgent then most likely to play safe, people will also send a patient to have formal ultrasound in the ultrasound department. So it won’t make much difference in patient management in the end. So it’s just the process. Maybe we’ll do one more thing and then convince the patient that you should go to A&E or do one more thing.” *(Participant 5- Public group practice*,* not interested in POCUS use*,* non-POCUS user*,* 19 years post HK grad)*.

## Discussion

To the best of our knowledge, this is the first mixed-methods study on the knowledge, attitudes, practices, and perceived barriers to using POCUS in primary care in Asia. We discovered that 22% of participants used POCUS, despite one-third having access to it; perceived knowledge was fair, and attitudes were mainly positive leading to high interest in POCUS use amongst the participants. However, we revealed a vicious cycle where participants felt inept in their POCUS knowledge and competence which was correlated with lower use of POCUS but barriers to training and limitations within their clinical practice prevented them from gaining the skills despite acknowledging the usefulness.

Globally, the prevalence of PCPs using POCUS varies from 1 to 47% in some European countries [3] to 76% in rural Canada [25]. The latter is higher because POCUS is particularly beneficial in rural areas that lack radiological support [26, 27]. Participants agreed that, in Hong Kong, timely access to private ultrasound imaging limits the need for POCUS.

We showed that perceived knowledge of POCUS is fair amongst participants. Knowledge relating to using POCUS and interpreting POCUS scored lower than knowledge of anatomy and indications on the use of POCUS. This finding could be explained because the former required more hands-on experience, but participants felt they lacked access to this experiential training. Additionally, we found that improved knowledge of POCUS was correlated to using POCUS in the past 12 months and interest in using POCUS, making perceived knowledge an important precursor for the adoption of POCUS use.

Attitudes for POCUS were generally positive similar to other international studies [11, 28], which likely led to the high level of interest in POCUS use. Most participants (92.4%) believed that POCUS was useful and would help to make earlier and more accurate diagnoses and assist with the management of patients, while also saving patients’ time, money, and effort, and unnecessary imaging studies and referrals to secondary care similar to findings in Western countries [28]. However, half of the respondents believe that radiologists should perform ultrasounds rather than PCP because the great majority of participants believe they lack the skills in using POCUS and that radiologists would be more skilled and make fewer errors. They also believed that documentation and reporting ultrasound findings were important but they lacked the skills. All of these combined heightened their anxieties about the risks of POCUS, such as missing diagnoses and medico-legal consequences. In contrast, Danish PCPs viewed POCUS differently from a radiologist’s ultrasound, understanding that their role was not to replicate the radiologist’s work [28]. This approach aligns with the teaching of POCUS in FM residency programs in the United States where PCPs are taught to use POCUS to answer a specific clinical question rather than doing an explorative scan of the area [18]. 

Of our respondents, 78.9% had positive attitudes regarding the cost-effectiveness of using POCUS similar to 71% in a Canadian study [14]. However, 70.5% stated financial concerns with POCUS use, not unlike other countries [3, 14]. Our qualitative data supported that some PCPs had financial barriers to equipment purchase, equipment maintenance, and training. Moreover, Hong Kong commercial real estate is one of the most expensive in the world, and few participants especially those already using POCUS were worried about the space larger (and higher-quality) machines would require, which is not a reported concern in other countries. However, there is also growing evidence showing that POCUS can lower costs by first employing it as a triage tool to determine which patients require more costly tests [9]. 

The majority of participants believed clinical and training support were also barriers, where the long time needed to train for POCUS and training course shortages were illustrated in qualitative interviews, similar to Western studies [3, 14]. A subset of participants wanted formal accreditation to protect POCUS users, but felt that the 2-year part-time Master’s program was too long. Despite the machine being available in many clinics, participants claimed the clinic had an inadequate number of trained staff to supervise. Qualitatively, consistently participants felt there seemed to be too few PCP POCUS trainers in Hong Kong, similar to Western countries [12, 14]. Lack of consultation time was another commonly reported barrier in other studies [3, 12]. The median time for POCUS use ranges from five to ten minutes [6, 7] which cannot fit in the average five to seven-minute consultation in Hong Kong public clinics [29]. Qualitatively, participants felt that potential costs existed with consultation time and POCUS use - either seeing fewer patients in a session or cutting out time that could be used for other aspects of the consultation. Participants, particularly those in the public sector, struggled to justify this opportunity cost though this cost could be transferred to patients in the private sector. In contrast, Danish general practitioners saw POCUS as an integrated part of the diagnostic process [28], but the average consultation time was ten to fifteen minutes [30]. Moreover, to combat this issue, few Hong Kong public primary care clinics have already started dedicated POCUS sessions for particular systems such as those for the heart and kidneys where specific PCPs would not only get more practice on a particular body part but would also have more time to spend with patients. In summary, we identified barriers that were unique to Asia. These included limited room for POCUS equipment, PCPs’ belief that radiologists are better at POCUS, and PCPs’ opinion that adding POCUS would mean trade-offs elsewhere.

Overall, there are potential benefits for POCUS in Hong Kong. POCUS has the potential to lower costs for patients by decreasing the necessity for private formal ultrasounds. Additionally, it can benefit the healthcare system by reducing referrals and improving health outcomes through earlier diagnoses. Training support for PCPs to overcome their lack of competency is needed, such as incorporating POCUS training into FM training programs, collaboration between HKCFP and the Hong Kong College of Radiologists, and joint POCUS clinics between PCPs and radiologists. Over time, more PCP POCUS trainers will be available to train the next generation of PCP POCUS users. Appropriate training on the purpose of utilizing POCUS in Hong Kong primary care (i.e. to address a clinical question rather than exploration while de-emphasizing the need for a formal report) may help with time management and also relieve fears about their competencies. Addressing consultation time constraints may require a top-down approach, where decision-makers overseeing doctor-patient quotas acknowledge the benefits of cost and time savings. Solutions may vary between the public and private sectors, with the public sector leaning towards specialized clinics as a favorable option. Concerns regarding limited space and cost may be mitigated as the technology and pricing for the hand-held machines improve and with more support from management. Future investigations into the barriers and facilitators to using POCUS in primary care at the organizational level can help explore how to deliver training and clinical assistance to the practitioners.

The study findings should be interpreted with caution considering the following limitations. Our study’s response rate was only 20.8%. However, it was comparable to other surveys distributed to PCPs in Hong Kong and the addition of the qualitative results improves the reliability of the results [31]. In addition, those who were more interested in the topic were more likely to fill in the survey and be willing to be interviewed, so the results may not be entirely generalizable to all PCPs in Hong Kong. However, for the interviews, we also selected PCPs who were not interested in POCUS to improve the balance.

## Conclusion

Although a majority of participants are interested and have positive attitudes toward POCUS, only about a quarter are using it. Overcoming the lack of competency entails providing training support for PCPs, whereas training on the purpose of utilizing POCUS may help with time management. A top-down approach with support from the managerial level may mitigate cost concerns. Future research should look at the organizational hurdles and enablers to employing POCUS in primary care to determine how realistically we can give physician training and clinical assistance.

## Supplementary Information

Supplementary Material 1.

Supplementary Material 2.

Supplementary Material 3.

Supplementary Material 4.

Supplementary Material 5.

Supplementary Material 6.

## Data Availability

The datasets used and/or analysed during the current study are available from the corresponding author on reasonable request.

## Abbreviations

FM: Family Medicine
HKCFP: The Hong Kong College of Family Physicians
PCP: Primary care physicians
POCUS: Point-of-care ultrasound
