# Supplementary material for: Knowledge, attitude, practices, and perceived barriers to using point-of-care ultrasound by Asian primary care physicians – a mixed method study
Source: BMC Health Serv Res. 2024 Nov 5;24:1344. doi: 10.1186/s12913-024-11865-5 (PMC11536830; doi:10.1186/s12913-024-11865-5)
Supplement: Supplementary file 1 — Supplementary Material 1. [file 12913_2024_11865_MOESM1_ESM.docx]

**Additional file 1 – POCUS in Primary Care Survey**

Thank you for agreeing to participate.

For the purpose of this survey, the term “*primary care*” will be used to describe outpatient practices in family medicine or general practice and excludes other specialties (such as emergency medicine, obstetrics and gynecology etc.)

The term “*point-of-care ultrasound” (POCUS)* will be used to describe ultrasound that will be carried out at the bedside by a portable ultrasound machine.

**Part I - Current practices of point-of-care ultrasound**

1. Does your current practice location haknowve access to an ultrasound facility that will provide a verbal and/or written report within 24 hours?

| ◻ Yes | ◻ No |
| --- | --- |

1. Does your current practice location have a POCUS device on-site?

| ◻ Yes | ◻ No | ◻ Not sure |
| --- | --- | --- |

1. Have you ever performed POCUS in primary care?

| ◻ Yes | ◻ No |
| --- | --- |

1. Have you used POCUS in the last 12 months in your clinical practice?

| ◻ Yes | ◻ No |
| --- | --- |

** online system will automatically skip question 5 if the response to number 4 is “No”

Check off all body systems or body parts that you have used POCUS on at least once in the past 12 months. Please also rate your competency, even if you have not used in the past 12 months.

Competency level is defined as

(1) Not competent: Do not adequately know the indications, anatomy, physiology or pathology of POCUS in this body system

(2) Mildly competent: Know the indications, anatomy, physiology and pathology of POCUS in this body system but not comfortable in performing and interpreting the findings of the ultrasound

(3) Quite competent: Know the indications, anatomy, physiology and pathology of POCUS in this body system and comfortable in performing and interpreting the findings of the ultrasound, but dependent on others for a need to cross check for ultrasound findings

(4) Very competent: Know the indications, anatomy, physiology and pathology of POCUS in this body system and comfortable in performing and interpreting the findings of the ultrasound independently

|  |  | **Competence Level** | | | |
| --- | --- | --- | --- | --- | --- |
|  | Used in past year at least once | Incompetent | Mildly Competent | Quite Competent | Very Competent |
| Arterial system (including Aorta) | ◻ | ◻ | ◻ | ◻ | ◻ |
| Breast | ◻ | ◻ | ◻ | ◻ | ◻ |
| Cardiac system (heart and valves) | ◻ | ◻ | ◻ | ◻ | ◻ |
| Gynecological system (excluding obstetrics) | ◻ | ◻ | ◻ | ◻ | ◻ |
| Hepatobiliary system | ◻ | ◻ | ◻ | ◻ | ◻ |
| Male genital system | ◻ | ◻ | ◻ | ◻ | ◻ |
| Musculoskeletal system | ◻ | ◻ | ◻ | ◻ | ◻ |
| Obstetrics | ◻ | ◻ | ◻ | ◻ | ◻ |
| Respiratory system | ◻ | ◻ | ◻ | ◻ | ◻ |
| Thyroid | ◻ | ◻ | ◻ | ◻ | ◻ |
| Urinary system | ◻ | ◻ | ◻ | ◻ | ◻ |
| Venous System | ◻ | ◻ | ◻ | ◻ | ◻ |
| Other: Please State ______ | ◻ | ◻ | ◻ | ◻ | ◻ |

**Part II – Attitudes to POCUS**

1. What is your attitude towards POCUS in the following statements?

|  | **Strongly disagree** | **Disagree** | **Agree** | **Strongly agree** |
| --- | --- | --- | --- | --- |
| Primary care doctors can be trained to use POCUS | ◻ | ◻ | ◻ | ◻ |
| POCUS in primary care can help to rule out certain conditions | ◻ | ◻ | ◻ | ◻ |
| There is sufficient evidence to prove that POCUS improves patient outcomes in the primary care setting | ◻ | ◻ | ◻ | ◻ |
| POCUS in primary care can increase diagnostic accuracy | ◻ | ◻ | ◻ | ◻ |
| POCUS in primary care can help to reduce referrals to hospitals or specialists | ◻ | ◻ | ◻ | ◻ |
| POCUS in primary care can allow for some procedures to be carried out more safely (ex. joint aspiration or injection) | ◻ | ◻ | ◻ | ◻ |
| POCUS in primary care can help to make further decisions on the need to order additional imaging investigations | ◻ | ◻ | ◻ | ◻ |
| POCUS training should be part of the family medicine vocational training | ◻ | ◻ | ◻ | ◻ |
| It is cost effective to use POCUS in primary care | ◻ | ◻ | ◻ | ◻ |
| Patients prefer ultrasound to be done by the radiology department rather than by their primary care physicians | ◻ | ◻ | ◻ | ◻ |
| POCUS used by primary care physicians could harm patients | ◻ | ◻ | ◻ | ◻ |

1. Would you be interested in using POCUS in your current practice?

| ◻ Yes | ◻ No | ◻ Not sure |  | ◻ Not applicable. I am already using POCUS |
| --- | --- | --- | --- | --- |

** online system - For those answering Yes, Not sure or Not Applicable move to question 8. Otherwise, if answer is “no” move to Section III)

1. Please state your level of interest for each body system or body part. For those who have already trained or are already using POCUS for that particular body part, please indicate if you would like further training.

|  | **Not interested at all** | **Not very interested** | **Somewhat interested** | **Very interested** |
| --- | --- | --- | --- | --- |
| Arterial system (including Aorta) | ◻ | ◻ | ◻ | ◻ |
| Breast | ◻ | ◻ | ◻ | ◻ |
| Cardiac system (heart and valves) | ◻ | ◻ | ◻ | ◻ |
| Gynecological system (excluding obstetrics) | ◻ | ◻ | ◻ | ◻ |
| Hepatobiliary system | ◻ | ◻ | ◻ | ◻ |
| Male genital system | ◻ | ◻ | ◻ | ◻ |
| Musculoskeletal system | ◻ | ◻ | ◻ | ◻ |
| Obstetrics | ◻ | ◻ | ◻ | ◻ |
| Respiratory system | ◻ | ◻ | ◻ | ◻ |
| Thyroid | ◻ | ◻ | ◻ | ◻ |
| Urinary system | ◻ | ◻ | ◻ | ◻ |
| Venous System | ◻ | ◻ | ◻ | ◻ |
| Other: Please State ______ | ◻ | ◻ | ◻ | ◻ |

**Part III – Barriers to POCUS**

1. Which of the following would you consider as important barriers to using POCUS in your practice? Please rate the following statements in terms of their importance as a BARRIER to using POCUS in your practice:

|  | **Not an important barrier at all** | **Not very important barrier** | **Somewhat important barrier** | **Very important barrier** |
| --- | --- | --- | --- | --- |
| Lack of confidence interpreting ultrasound images without having a radiologist available to confirm them | ◻ | ◻ | ◻ | ◻ |
| Lack of usefulness of POCUS to my specific clinical practice | ◻ | ◻ | ◻ | ◻ |
| Lack of formal accreditation in POCUS in Hong Kong | ◻ | ◻ | ◻ | ◻ |
| Lack of support from the clinic to perform POCUS (such as lack of supervision) | ◻ | ◻ | ◻ | ◻ |
| Lack of time to use POCUS during the consultation | ◻ | ◻ | ◻ | ◻ |
| Lack of access to POCUS devices in the clinic | ◻ | ◻ | ◻ | ◻ |
| Lack of time to train for POCUS | ◻ | ◻ | ◻ | ◻ |
| Lack of available training courses for POCUS | ◻ | ◻ | ◻ | ◻ |
| Lack of financial reimbursement when performing POCUS | ◻ | ◻ | ◻ | ◻ |
| Cost of purchasing POCUS would be too much | ◻ | ◻ | ◻ | ◻ |
| Cost of training courses for POCUS would be too much | ◻ | ◻ | ◻ | ◻ |
| Possible steep learning curve | ◻ | ◻ | ◻ | ◻ |
| Possible litigation problems with POCUS | ◻ | ◻ | ◻ | ◻ |

**Part IV –Perceived knowledge of POCUS**

1. How would you rate your current level of knowledge or skill in the following domains? If you already use POCUS, please answer based on your knowledge of body systems for which you use POCUS. If you do not use POCUS, please answer based on any body system for which you are interested in using POCUS. (*Note a rating of “Excellent” should be at a level similar with a radiologist specialist)

|  | **Inadequate** | **Fair** | **Good** | **Excellent** |
| --- | --- | --- | --- | --- |
| Knowledge of the indications for POCUS | ◻ | ◻ | ◻ | ◻ |
| Knowledge in anatomy | ◻ | ◻ | ◻ | ◻ |
| Knowledge of pathology | ◻ | ◻ | ◻ | ◻ |
| Knowledge of the physics of ultrasound |  |  |  |  |
| Knowledge of using the ultrasound probe and machine | ◻ | ◻ | ◻ | ◻ |
| Knowledge of choosing different modes of imaging | ◻ | ◻ | ◻ | ◻ |
| Knowledge of documentation of POCUS image and report | ◻ | ◻ | ◻ | ◻ |
| Knowledge in interpretation of ultrasound images | ◻ | ◻ | ◻ | ◻ |

**Part VI - Basic Demographic Information**

1. What is your age? ____ * online use drop down menu
2. What is your gender?

| ◻ Male | ◻ Female | ◻ Prefer not to say |
| --- | --- | --- |

1. What year did you graduate from medical school? ______ *online use drop down menu
2. Which country did you graduate from?

| ◻ Hong Kong | ◻ Outside of Hong Kong.  Please provide the country _______ |
| --- | --- |

1. What are your post-graduate qualifications? (select all that applies)

| ◻ FRACGP | ◻ FHKAM (Family Medicine) |
| --- | --- |
| ◻ Diploma in Family Medicine | ◻ FHKCFP |
| ◻ Currently undergoing basic training in Family Medicine | ◻ Other ___________ |
| ◻ Currently undergoing higher training in Family Medicine |  |

1. During the last 12 months, were you ever in-practice as a general practitioner or a family physician or undergoing vocational training in Family Medicine?

| ◻ Yes | ◻ No |
| --- | --- |

1. What service institution are you currently working at?

| ◻ Solo private clinic | ◻ Department of Health |
| --- | --- |
| ◻ Hospital Authority | ◻ Group private practice |
| ◻ University health services | ◻ Private hospital |
| ◻ Not practicing | ◻Other |

1. What type of training have you had in POCUS and how many hours of training did you receive? (please input “0” under the number of hours if you have not trained in that particular modality)

|  |  | Number of hours spent |
| --- | --- | --- |
| 20a) | ◻ Master of Science in Diagnostic Ultrasound | _________ |
| 20b) | ◻ Hands-on workshops | _________ |
| 20c) | ◻ Didactic seminar | _________ |
| 20d) | ◻ Bedside teaching | _________ |
| 20e) | ◻ Others: (Please specify) _______ | _________ |

1. What is the last 4 digits of your mobile phone number? (Please note: this is only used to ensure that there are no duplicate responses but will not be used for any other purposes and cannot be traced back directly to you.)

__ ___ ___ ___
