# Supplementary material for: Knowledge, attitude, practices, and perceived barriers to using point-of-care ultrasound by Asian primary care physicians – a mixed method study
Source: BMC Health Serv Res. 2024 Nov 5;24:1344. doi: 10.1186/s12913-024-11865-5 (PMC11536830; doi:10.1186/s12913-024-11865-5)
Supplement: Supplementary file 2 — Supplementary Material 2. [file 12913_2024_11865_MOESM2_ESM.docx]

**Additional file 2 – Interview guide**

Code: Date: Time: Venue:

**Introduction**

We are here to talk about point-of-care ultrasonography also known as bedside ultrasound today. This study examines the knowledge, attitudes, practices and the perceived barriers of using bedside ultrasound in primary care physicians in Hong Kong. I will be asking a series of questions related to this, in the hopes to learn more about how primary care physicians really think regarding this investigation.

Participation is completely voluntary. If there are any questions that you do not want to answer. The expected length of this interview will be up to 45 minutes.

We will be recording the session so that we are able to transcribe the interview and get accurate quotes from our participants. Your name will never be associated with anything we may publish or present in the future about this, so please ask freely, openly and honestly as possible. I would invite you at this time to rename yourself as “Participant #” and turn off your camera.

Would you be comfortable to proceed with the interview?

[IF affirmative answer, the recording will begin and will go into the first question]

| **Topic Guide** |
| --- |
| *Can you tell me what happens at your usual practice when you want to order an ultrasound?* |
| *What factors are you taking into consideration whether or not to use POCUS in your practice?* |
| *What factors influence your interest in using bedside ultrasound?* |
| *What benefits do you see with using bedside ultrasound in family practice?* |
| *What barriers do you have that prevent you from using bedside ultrasound (or from using bedside ultrasound more often)?* |
| *Do you have any suggestions on how to improve the barriers to using bedside ultrasound?* |
