# Supplementary material for: Knowledge, attitude, practices, and perceived barriers to using point-of-care ultrasound by Asian primary care physicians – a mixed method study
Source: BMC Health Serv Res. 2024 Nov 5;24:1344. doi: 10.1186/s12913-024-11865-5 (PMC11536830; doi:10.1186/s12913-024-11865-5)
Supplement: Supplementary file 3 — Supplementary Material 3. [file 12913_2024_11865_MOESM3_ESM.docx]

**Categorization of subgroups for domains “attitude” and “barriers”**

| **Variables** | **Subgroups** | **Inclusion of statements** |
| --- | --- | --- |
| Attitude | On POCUS Training | - Primary care doctors can be trained to use POCUS. - POCUS training should be part of the family medicine vocational training. |
|  | On clinical usefulness of POCUS | - POCUS in primary care can help to rule out certain conditions. - There is sufficient evidence to prove that POCUS improves patient outcomes in the primary care setting. - POCUS in primary care can increase diagnostic accuracy. - POCUS in primary care can help to reduce referrals to hospitals or specialists. - POCUS in primary care can allow for some procedures to be carried out more safely (ex. joint aspiration or injection). - POCUS in primary care can help to make further decision on the need to order additional imaging investigations. |
|  | On harmfulness of POCUS | - POCUS used by primary care physicians could harm patients. |
|  | On cost-effectiveness of POCUS | - It is cost effective to use POCUS in primary care. |
|  | On patient preference on provider | - Patients prefer ultrasound to be done by the radiology department rather than by their primary care physicians. |
| Barriers | Competence of POCUS skills | - Lack of confidence interpreting ultrasound images without having a radiologist available to confirm them. - Possible steep learning curve. - Possible litigation problems with POCUS. |
|  | Clinical usefulness | - Lack of usefulness of POCUS to my specific clinical practice. |
|  | Training support | - Lack of formal accreditation in POCUS in Hong Kong. - Lack of time to train for POCUS. - Lack of training courses for POCUS. |
|  | Clinical support | - Lack of support from the clinic to perform POCUS (such as lack of supervision). - Lack of time to use POCUS during the consultation. - Lack of access to POCUS devices in the clinic. |
|  | Financial concerns | - Lack of financial reimbursement when performing POCUS. - Cost of purchasing POCUS would be too much. - Cost of training courses for POCUS would be too much. |
