# Supplementary material for: Knowledge, attitude, practices, and perceived barriers to using point-of-care ultrasound by Asian primary care physicians – a mixed method study
Source: BMC Health Serv Res. 2024 Nov 5;24:1344. doi: 10.1186/s12913-024-11865-5 (PMC11536830; doi:10.1186/s12913-024-11865-5)
Supplement: Supplementary file 4 — Supplementary Material 4. [file 12913_2024_11865_MOESM4_ESM.docx]

**Descriptive analysis for each survey item under domain “knowledge”, “attitude” and “barriers” by Likert scale category**

|  | **Likert scale** | | | |
| --- | --- | --- | --- | --- |
|  | 1 | 2 | 3 | 4 |
| **Knowledge ^1^** |  |  |  |  |
| Knowledge of the indications for POCUS | 70 (25.5%) | 99 (36.0%) | 93 (33.8%) | 13 (4.7%) |
| Knowledge in anatomy | 55 (20.0%) | 155 (56.4%) | 62 (22.5%) | 3 (1.1%) |
| Knowledge of pathology | 66 (24.0%) | 146 (53.1%) | 60 (21.8%) | 3 (1.1%) |
| Knowledge of the physics of ultrasound | 119 (43.3%) | 105 (38.2%) | 44 (16.0%) | 7 (2.5%) |
| Knowledge of using the ultrasound probe and machine | 117 (42.5%) | 104 (37.8%) | 45 (16.4%) | 9 (3.3%) |
| Knowledge of choosing different modes of imaging | 120 (43.6%) | 97 (35.3%) | 51 (18.5%) | 7 (2.5%) |
| Knowledge of documentation of POCUS image and report | 137 (49.8%) | 101 (36.7%) | 34 (12.4%) | 3 (1.1%) |
| Knowledge in interpretation of ultrasound images | 151 (54.9%) | 89 (32.4%) | 32 (11.6%) | 3 (1.1%) |
| **Attitude ^2^** |  |  |  |  |
| Primary care doctors can be trained to use POCUS | 8 (2.9%) | 6 (2.2%) | 160 (58.2%) | 101 (36.7%) |
| POCUS in primary care can help to rule out certain conditions | 7 (2.5%) | 10 (3.6%) | 168 (61.1%) | 90 (32.7%) |
| There is sufficient evidence to prove that POCUS improves patient outcomes in the primary care setting | 8 (2.9%) | 30 (10.9%) | 184 (66.9%) | 53 (19.3%) |
| POCUS in primary care can increase diagnostic accuracy | 6 (2.2%) | 13 (4.7%) | 200 (72.7%) | 56 (20.4%) |
| POCUS in primary care can help to reduce referrals to hospitals or specialists | 8 (2.9%) | 34 (12.4%) | 172 (62.5%) | 61 (22.2%) |
| POCUS in primary care can allow for some procedures to be carried out more safely | 4 (1.5%) | 18 (6.5%) | 165 (60.0%) | 88 (32.0%) |
| POCUS in primary care can help to make further decisions on the need to order additional imaging investigations | 6 (2.2%) | 15 (5.5%) | 187 (68.0%) | 67 (24.4%) |
| POCUS training should be part of the family medicine vocational training | 14 (5.1%) | 46 (16.7%) | 150 (54.5%) | 65 (23.6%) |
| It is cost effective to use POCUS in primary care | 6 (2.2%) | 52 (18.9%) | 171 (62.2%) | 46 (16.7%) |
| Patients prefer ultrasound to be done by the radiology department rather than by their primary care physicians | 27 (9.8%) | 106 (38.5%) | 132 (48.0%) | 10 (3.6%) |
| POCUS used by primary care physicians could harm patients | 4 (1.5%) | 54 (19.6%) | 172 (62.5%) | 45 (16.4%) |
| **Barriers ^3^** |  |  |  |  |
| Lack of confidence interpreting ultrasound images without having a radiologist available to confirm them | 4 (1.5%) | 15 (5.5%) | 116 (42.2%) | 140 (50.9%) |
| Lack of usefulness of POCUS to my specific clinical practice | 43 (15.6%) | 121 (44.0%) | 93 (33.8%) | 18 (6.5%) |
| Lack of formal accreditation in POCUS in Hong Kong | 13 (4.7%) | 47 (17.1%) | 125 (45.5%) | 90 (32.7%) |
| Lack of support from the clinic to perform POCUS | 6 (2.2%) | 24 (8.7%) | 114 (41.5%) | 131 (47.6%) |
| Lack of time to use POCUS during the consultation | 8 (2.9%) | 35 (12.7%) | 90 (32.7%) | 142 (51.6%) |
| Lack of access to POCUS devices in the clinic | 19 (6.9%) | 54 (19.6%) | 101 (36.7%) | 101 (36.7%) |
| Lack of time to train for POCUS | 9 (3.3%) | 34 (12.4%) | 115 (41.8%) | 117 (42.5%) |
| Lack of available training courses for POCUS | 2 (0.7%) | 2 (7.6%) | 2 (41.1%) | 2 (50.5%) |
| Lack of financial reimbursement when performing POCUS | 18 (6.5%) | 73 (26.5%) | 110 (40.0%) | 74 (26.9%) |
| Cost of purchasing POCUS would be too much | 21 (7.6%) | 77 (28.0%) | 99 (36.0%) | 78 (28.4%) |
| Cost of training courses for POCUS would be too much | 12 (4.4%) | 69 (25.1%) | 131 (47.6%) | 63 (22.9%) |
| Possible steep learning curve | 8 (2.9%) | 73 (26.5%) | 140 (50.9%) | 54 (19.6%) |
| Possible litigation problems with POCUS | 9 (3.3%) | 35 (12.7%) | 116 (42.2%) | 115 (41.8%) |

^1^ Likert scale score 1, 2, 3, and 4 refer to option "strongly inadequate", "fair", "good" and "excellent" respectively

^2^ Likert scale score 1, 2, 3, and 4 refer to option "strongly disagree", "disagree", "agree" and "strongly agree" respectively

^3^ Likert scale score 1, 2, 3, and 4 refer to option "not an important barrier at all", "not very important barrier", “somewhat important barrier", and "very important barrier" respectively
