# Supplementary material for: Knowledge, attitude, practices, and perceived barriers to using point-of-care ultrasound by Asian primary care physicians – a mixed method study
Source: BMC Health Serv Res. 2024 Nov 5;24:1344. doi: 10.1186/s12913-024-11865-5 (PMC11536830; doi:10.1186/s12913-024-11865-5)
Supplement: Supplementary file 5 — Supplementary Material 5. [file 12913_2024_11865_MOESM5_ESM.docx]

**Logistic regression on “knowledge”, “attitude”, “interest” and “usage” for “interest” and “usage” of respondents**

|  | **Interested in POCUS  (N=275)** | |  | **Used POCUS in the last 12 months in their clinical practice (N=275)** | |
| --- | --- | --- | --- | --- | --- |
|  | Odd Ratio (95% C.I.) | P-value |  | Odd Ratio (95% C.I.) | P-value |
| **Knowledge** | 1.115 (0.654, 1.901) | 0.689 |  | 6.511 (3.517, 12.053) | <0.001* |
| **Attitude** |  |  |  |  |  |
| On POCUS Training - Positive | 4.086 (1.897, 8.8) | <0.001* |  | 0.832 (0.309, 2.24) | 0.715 |
| On clinical usefulness of POCUS - Positive | 2.315 (0.625, 8.578) | 0.209 |  | 2.01 (0.455, 8.878) | 0.357 |
| On harmfulness of POCUS - Positive | 1.899 (0.896, 4.026) | 0.094 |  | 0.821 (0.329, 2.053) | 0.674 |
| On cost-effectiveness of POCUS - Positive | 1.484 (0.783, 2.813) | 0.226 |  | 2.057 (0.968, 4.37) | 0.061 |
| On patient preference on provider - Positive | 2.119 (0.942, 4.765) | 0.069 |  | 0.404 (0.154, 1.064) | 0.067 |
| **Interest** in using POCUS in current practice | NA | NA |  | 0.735 (0.327, 1.653) | 0.457 |
| **Practice** |  |  |  |  |  |
| Used POCUS in the last 12 months in their clinical practice | 0.677 (0.303, 1.514) | 0.342 |  | NA | NA |

NA=Not applicable

Notes:

Odd ratio was also controlled for gender, years after graduation from medical school, country of graduation, and type of service institutions.

*Significance at p-value < 0.05
