# Supplementary material for: Knowledge, attitude, practices, and perceived barriers to using point-of-care ultrasound by Asian primary care physicians – a mixed method study
Source: BMC Health Serv Res. 2024 Nov 5;24:1344. doi: 10.1186/s12913-024-11865-5 (PMC11536830; doi:10.1186/s12913-024-11865-5)
Supplement: Supplementary file 6 — Supplementary Material 6. [file 12913_2024_11865_MOESM6_ESM.docx]

**Additional quotes on the subthemes of qualitative data**

| **Themes/Subthemes** | | **Example Quotes** |
| --- | --- | --- |
| **Theme 1 – Usefulness of POCUS** | | |
| 1. Improving diagnosis and management | | There are treatment modalities that can be enhanced by ultrasound guidance like ultrasound guided joint injections, ultrasound guided nerve blocks or any... procedures that we might do. *(Participant 3 – Private group, non-POCUS user, interested in POCUS use, 13 years post UK grad)* |
| 1. Reducing burdens on patients | | Ultrasound can be a useful bedside test compared with X-ray… they can use the ultrasound and avoid radiation of the X rays. *(Participant 8 – Public group, POCUS user, >10 years post HK grad)* |
| 1. Reducing burdens on the healthcare system | | We find out a problem after taking the bedside scan, and we don’t need to redo an ultrasound in the next step. We can order a further investigation right away. *(Participant 10 – Private solo, POCUS user, 26 years post HK grad)* |
|  | | |
| **Theme 2 – Competence of POCUS skill** | | |
| 1. Encountering barriers for training | | I think [time needed to train for POCUS] is the biggest problem up to this moment, because… there is an ultrasound course in [Chinese University of Hong Kong], but it’s a 2-year master course, so the time investment is too much. It’s a huge barrier. *(Participant 10 – Private solo, POCUS user, 26 years post HK grad)*  So if we... can have a formal training, like by the [College of Family Physicians], we can lay some credentialing framework or training framework, that will be good... Because it also helps to protect those who are using POCUS. For example, when something bad happens, … the MCHK [Medical Council of Hong Kong] will look at your guidelines and whether your College have any documents, guidelines, frameworks for credentials, or for trainings, the competency levels. *(Participant 6 – Public group, POCUS user, 19 years post HK grad)* |
| 1. Lacking confidence for competent POCUS skills and its related problems | | Well, obviously, my own competency in operating the ultrasound machine [and] interpreting the image… would be my first priority. To be frank, I don’t think I am competent in doing an ultrasonic patient because it’s really not... being well covered in my medical education. *(Participant 5 – Public group, non-POCUS user, not interested in POCUS use, 19 years post HK grad)* |
| 1. Radiologists are better skilled at ultrasound | One of the reasons that we... don’t have .... formal ultrasound reports is that we are not specialists. I mean we are not radiologists and we cannot write a formal report for that. *(Participant 11 – Public group, non-POCUS user, interested in POCUS use, higher trainee, 6 years post HK grad)* | |
|  | | |
| **Theme 3 – Limitations in using POCUS in clinical practice** | | |
| 1. Time to use POCUS during the consultation | Your consultation is like 15 to 20 minutes max. If you did an ultrasound, that will take you like, maybe like 10 minutes. So, you either cut short your other bits of the consultation or then you will overrun. *(Participant 14 – Private hospital, non-POCUS user, interested in POCUS use, 9 years post HK grad )* | |
| 1. Concerns on POCUS machine | I imagine purchasing the ultrasound machine now, talking about HK$740,000 which is equipped with cardiac imaging function. So that is [going to be] a long process... To get this machine, we need to have a project, so we submit a proposal, we bid for charity funding and finally, we get the funding… around one year time already. *(Participant 9 – Public group, POCUS user, 20 years post HK grad)* | |
| 1. No compelling need for POCUS in current practice | I mean I can’t speak for private practice in Hong Kong, but in the [General Outpatient Clinic] setting, the bulk of one’s time is taken up with chronic disease management, so hypertension, diabetes, hyperlipidemia. And so ultrasound is not so specifically relevant to these conditions. *(Participant 4 – Private group, non-POCUS user, interested in POCUS use, 19 years post HK Grad)* | |
